# Developing NiAl-Strengthened ULCB Steels by Controlling Nanoscale Precipitation and Reversed Austenite

**DOI:** 10.3390/ma18122822

**Published:** 2025-06-16

**Authors:** Jize Guo, Xiyang Chai, Shuo Gong, Zemin Wang, Tao Pan

**Affiliations:** 1 Division of Structural Steels, Central Iron and Steel Research Institute, Beijing 100081, China; 13546856584@163.com (J.G.); chaixiyang0728@163.com (X.C.); gongshuodd@163.com (S.G.); 2 School of Materials Science and Engineering, Shanghai Institute of Technology, 100 Haiquan Road, Shanghai 201418, China; wzm@sit.edu.cn

**Keywords:** ultra-low carbon bainite, nanoscale precipitation, TMCP, tempering

## Abstract

In this study, a strategy was adopted to promote the formation of NiAl precipitates with the aim of enhancing strength by incorporating a 0.2 wt.% Al into a traditional ultra-low carbon bainitic (ULCB) steel alloy. By integrating thermo-mechanical control processing (TMCP) and a tailored tempering process, a new-generation steel with an outstanding combination of properties has been successfully developed for shipbuilding and marine engineering equipment. It features a yield strength of 880 MPa, a yield ratio of 0.84, and an impact toughness of 175 J. The precipitation characteristics of nanoscale particles in this steel, including NiAl intermetallics and carbides, were systematically investigated. The results show that the alloy with low Al addition formed NiAl precipitates during tempering. The high-density distributions of NiAl, (Mo, V)C, and (Ti, V, Nb)C precipitates, which exhibit slow coarsening kinetics, played a dominant role in enhancing the strength of the tempered steel. In addition to precipitation, the microstructure before and after tempering was also analyzed. It was observed that a granular bainite morphology was favorable for decreasing the yield ratio. Additionally, the formation of reverse-transformed austenite during tempering was critical for retaining toughness despite substantial strength gains. Finally, theoretical modeling was employed to quantitatively assess the contributions of these microstructural modifications to yield strength enhancement of thermo-mechanical controlled processing (TMCP) and tempered steel. This study establishes a fundamental basis for subsequent industrial-scale development and practical engineering applications of novel products.

## 1. Introduction

The rapid development of the maritime economy has created significant opportunities for technical advances in ships and offshore engineering equipment [1]. The increasing demands for the large-scale, high-efficiency manufacturing and safe service performance of marine structures require structural materials with enhanced strength, weldability, and long-term reliability [2]. Current mainstream marine engineering steels [3] can be categorized into three groups: (1) low-alloy high-strength steels (e.g., DH-36 [4] and EH36 [5]), which exhibit a ferrite–pearlite microstructure that limits their maximum strength to approximately 500 MPa; (2) HY-series steels with tempered martensite microstructures, offering favorable strength and toughness, although their high carbon equivalents place them in the non-weldable zone of the Graville diagram [6], resulting in poor weldability; and (3) high-strength low-alloy (HSLA) steels, designed with reduced carbon content to improve weldability and copper additions for precipitation strengthening. Nevertheless, HSLA steels exhibit yield ratios exceeding 0.9, leading to compromised safety performance during service.

In recent years, alloying strategies involving aluminum have provided a new pathway for developing advanced marine steels [7,8]. The B2-NiAl phase precipitates as the coherent nanoscale particles (<5 nm) due to its lattice parameter matching that of BCC ferrite, thereby providing significant strengthening effects [9,10]. Jiao et al. [10] achieved high-number-density NiAl intermetallic precipitates through precise optimization of Ni and Al contents, expanding the application scope of intermetallic strengthening phases. Furthermore, Jiang et al. [11] leveraged the strengthening mechanism of NiAl phases to design and fabricate ultrahigh-strength (2 GPa) steels containing nanoscale precipitates with high number density and low lattice misfit.

However, the inadequate low-temperature impact toughness of NiAl-strengthened steels has restricted their extended applications in marine engineering fields, and the issues of ductility and yield ratio in NiAl-strengthened steel have been rarely mentioned. Previous studies have shown that the toughness of many NiAl-strengthened steels containing martensitic structure decreases obviously after tempering process [12]. The toughness of steel is related to the microstructure of matrix and the multiphase structure in ULCB steel can effectively enhance toughness by reverse austenite formed during the tempering process. [13]. Additionally, the yield ratios of ULCB steels can be effectively reduced by optimizing the ratio of bainitic and ferrite to martensite–austenite (M/A) islands [14,15]. Nevertheless, the microstructural features and the NiAl precipitate states in ULCB steels required to achieve the desired properties during the tempering process have not been carefully identified.

The purpose of this study is to investigate how the tempering temperature influences the mechanical properties of the steel through adjusting the precipitation states of the NiAl phase and the morphologies of reverse austenite. A novel alloy of NiAl-strengthened steel with 7 wt.% Ni and 0.2 wt.% Al was designed. By integrating thermo-mechanical control processing (TMCP) and a tailored tempering process, the study achieved the coupling of NiAl phase strengthening, reverted austenite toughening, and the low yield ratio characteristic of the granular bainite matrix, thereby realizing an excellent combination of 880 MPa yield strength, 0.84 yield ratio, and 175 J impact toughness.

## 2. Methods

### 2.1. Production of the Experimental Alloy

**Figure 1:**
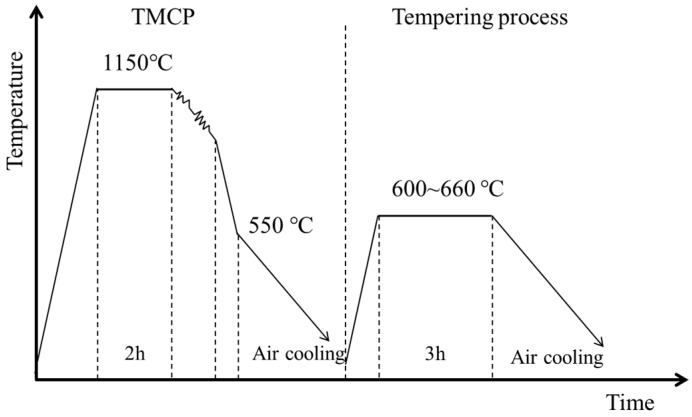
TMCP and heat treatment procedure.

A total of 200 kg of experimental steel was produced via vacuum induction melting and forged into a billet (270 × 210 × 60 mm^3^). The chemical composition of the experimental steel is shown in Table 1. The heating process parameters were determined as 1150 °C for 2 h to achieve full dissolution of precipitates and homogenization of the matrix in the experimental steel. Based on analysis of the continuous cooling transformation (CCT) curve, where the bainite transformation start temperature exceeded 446 °C, the final cooling temperature was set to 550 °C to obtain a granular bainitic microstructure combining high strength, toughness, and low yield ratio. The experimentally measured Ac1 temperature was 661 °C. To investigate the optimal tempering temperature for achieving balanced mechanical properties, multiple tempering temperatures below the Ac1 phase transformation point were selected. This design enabled concurrent precipitation strengthening of NiAl intermetallic compounds and toughness regulation through reverted austenite formation during the tempering process. The complete processing flowchart of the experimental steel is systematically presented in Figure 1. The forged billets were subsequently reheated to 1150 °C, held at this temperature for 2 h, and then hot-rolled into 30 mm thick plates through multiple passes. The finished hot-rolling temperature was approximately 800 °C. Finally, the billets were tempered at 600, 620, 640, and 660 °C for 3 h and then air-cooled to room temperature.

### 2.2. Mechanical Characterization of Steel

Mechanical property test specimens and microstructural characterization samples were extracted along the transverse direction of the rolled plate. For each processing condition, two tensile specimens with dimensions of M16 × Φ10 mm were prepared. Room-temperature tensile testing was conducted using a GNT-300 universal testing machine in compliance with GB/T228.1-2021 standard [16], employing a constant crosshead speed of 2 mm/min and an extensometer gauge length of 50 mm. Six transverse V-notch Charpy impact specimens (10 × 10 × 55 mm^3^) were fabricated per processing condition. These specimens were tested at −20 °C and −84 °C using an ELYI02 pendulum impact tester (three replicates per temperature) according to GB/T229-2020 standard [17], with average values calculated from the experimental results. Instrumented impact testing was specifically performed at −84 °C to record the force–displacement–energy curves of the experimental steel.

### 2.3. Characterization of the Experimental Alloy

Metallographic specimens were ground, polished, and etched with 10 vol% nitric acid alcohol solution. Microstructural characterization was performed using a field-emission scanning electron microscope (SEM, Quanta-650, FEI, Carl Zeiss, Inc., Hillsboro, OR, USA) in secondary electron mode with an accelerating voltage of 20 kV. Fine structural analysis was conducted via a FEI Talos F200X transmission electron microscope (TEM) operated at 200 kV, equipped with an energy-dispersive spectrometer (EDS, Bruker, Karlsruhe, Germany) for line-scan elemental analysis. TEM specimen preparation involved mechanical thinning to 50 μm thickness, followed by punching into 3 mm diameter discs, which were subsequently electropolished using a Struers TenuPol-5 twin-jet polisher (Struers, Ballerup, Denmark) with 10 vol% perchloric acid alcohol electrolyte maintained at −25 °C.

Three-dimensional atom probe tomography (APT) was conducted on a Cameca LEAP 4000X SI instrument (Cameca, Gennevilliers, France) to characterize the chemical composition of nanostructures. Specimens were analyzed under a base temperature of 40 K, with pulsed laser parameters set at 50 pJ energy and 200 kHz frequency, maintaining a target evaporation rate of 1.0% per pulse. APT data reconstruction and statistical analysis were performed using Cameca IVAS 3.6.12 software. The mean radius (R) and number density (N) of the nanoparticles were calculated using the cluster method, as described in [18]. Needle-shaped APT specimens were prepared through a standard two-step electrochemical polishing procedure.

The Mn-Ni-Al ULCB steel samples were cut with a surface area of 10 × 10 × 5 mm, sandpapered, and mechanically polished, and X-ray diffraction (XRD) analysis was performed. XRD analysis was performed on a D8 ADVANCE diffractometer (Bruker, Karlsruhe, Germany) with Co-Kα radiation (40 mA, 35 kV) at a scanning rate of 2°/min and a scanning angle (2θ) range of 45–115° to determine dislocation density. XRD data were processed using HighScorePlus 3.0.5 software with PDF card reference for phase identification. The full width at half maximum (FWHM) of α-Fe diffraction peaks was extracted for dislocation density estimation via the modified Williamson–Hall (MWH) method.

## 3. Results

### 3.1. Mechanical Properties

**Figure 2:**
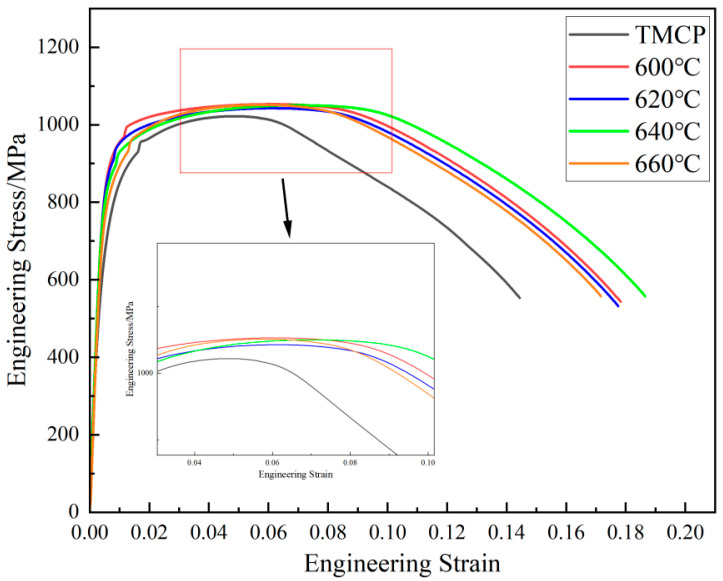
The stress–strain curves of the experimental steels.

**Figure 3:**
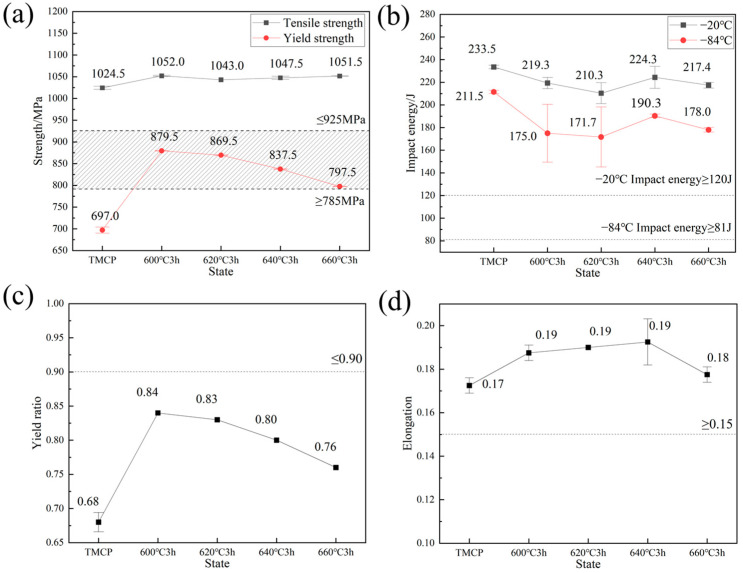
Effects of tempering temperature on the mechanical properties of the experimental steel. (**a**) Strength. (**b**) Impact energy. (**c**) Yield ratio. (**d**) Elongation.

The stress–strain curves of the experimental steel under TMCP and tempered conditions are presented in Figure 2, with the mechanical properties summarized in Figure 3, with dashed lines indicating the target performance criteria. Tempering at 600 °C significantly improved the yield strength and yield ratio of the TMCP-processed steel, while reducing its toughness. Specifically, the yield strength increased from 697 MPa to 879.5 MPa, and the yield ratio rose from 0.68 to 0.84. Concurrently, the impact energy decreased from 233.5 J to 219.3 J at −20 °C and from 211.5 J to 172 J at −84 °C. As the tempering temperature increased from 600 °C to 660 °C, the yield strength gradually declined from 879.5 MPa to 797.5 MPa, whereas the tensile strength remained stable within the range of 1043–1052 MPa. This indicates that the reduction in yield ratio during tempering was primarily attributed to the decrease in yield strength. Notably, the tempering temperature exhibited limited influence on impact energy: the −20 °C impact energy of tempered steel ranged between 210.3 J and 224.3 J, while the −84 °C impact energy varied slightly from 171.7 J to 190.3 J.

### 3.2. Microstructure

**Figure 4:**
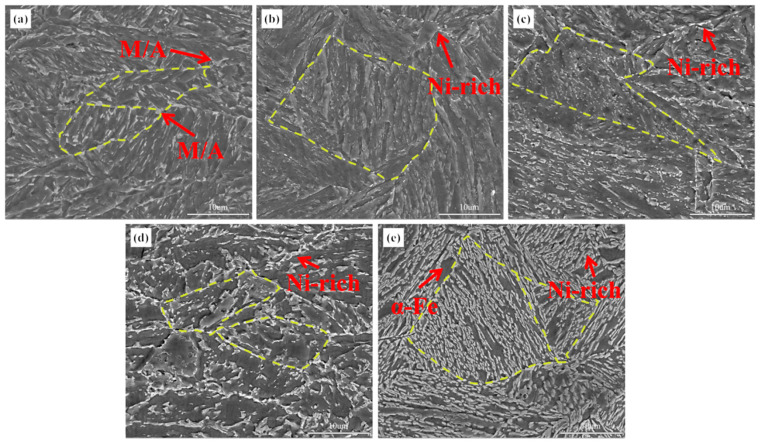
Microstructure of the experimental steel at different states. (**a**) TMCP state; (**b**) 600 °C; (**c**) 620 °C; (**d**) 640 °C; (**e**) 660 °C.

Figure 4 shows the SEM images of the experimental steel in the TMCP condition and after tempering at different temperatures. The TMCP-processed steel exhibited a granular bainite microstructure, primarily composed of bainitic ferrite and M/A islands (Figure 4a). The M/A islands were predominantly located at prior austenite grain boundaries and between bainitic ferrite laths. Upon tempering at 600 °C, the recovery of the bainitic ferrite laths occurred, accompanied by the partial decomposition of M/A islands. Bright white elemental-enriched zones emerged, while residual M/A islands remained distributed between the bainitic ferrite laths, indicating the transformation of granular bainite into tempered granular bainite. With the tempering temperature increasing to 620 °C and 640 °C, a significant recovery and coarsening of the bainitic ferrite laths were observed. The lath bundles gradually merged, leading to the disappearance of their characteristic boundaries (Figure 4c,d). Concurrently, there was further decomposition of M/A islands and an increased number and larger size of bright white regions along prior grain boundaries. At 660 °C tempering, approaching the two-phase region (Ac1 = 661 °C), the microstructure displayed pronounced elemental enrichment. Bright white zones expanded extensively, and ferrite formation was detected in localized areas.

**Figure 5:**
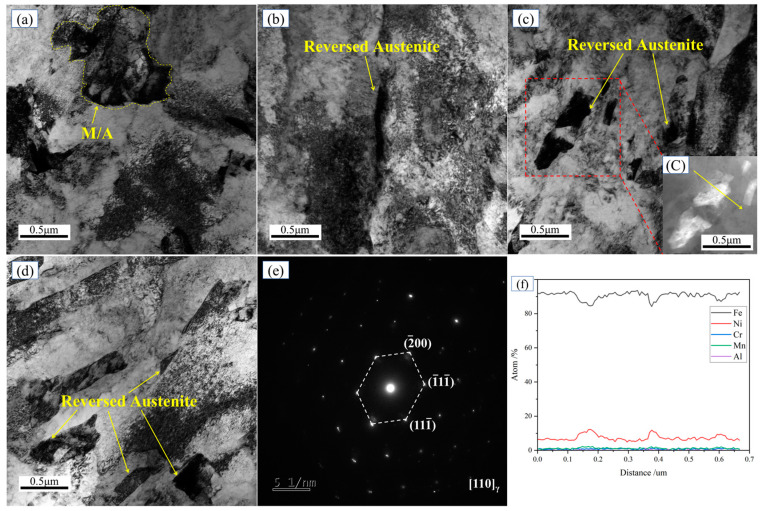
TEM characterization of austenite in experimental steel tempered at different states. (**a**) TMCP state; (**b**) 600 °C; (**c**) 620 °C; (**C**) Partial enlarged view of reverted austenite; (**d**) 640 °C; (**e**) SAED of reversed austenite; (**f**) line scan of reversed austenite.

Figure 5 presents the TEM images of the experimental steel in the TMCP condition and after tempering at different temperatures. In the TMCP-processed steel, austenite primarily existed in the form of M/A islands (Figure 5a). Following tempering at 600 °C, lamellar reversed austenite formed at grain boundaries. With increasing tempering temperature, the reversed austenite underwent coarsening and morphological changes. After tempering at 640 °C, the reversed austenite at grain boundaries predominantly exhibited a blocky morphology (Figure 5d).

The selected-area diffraction (SAED) pattern from the 620 °C tempered steel (Figure 5e) was indexed to the [110] zone axis of face-centered cubic (FCC) austenite. A line scan across the reversed austenite (inset in Figure 5c) revealed significant Ni enrichment (14%) compared to the matrix (~7%), as shown in Figure 5f. These results confirm that the bright regions in the SEM images correspond to reversed austenite. Ni, acting as an austenite-stabilizing element, segregated at grain boundaries during tempering, thereby reducing the local austenitization temperature and enabling the formation of austenite prior to entering the two-phase region. Acting as a soft phase, the reversed austenite enhanced toughness by impeding crack propagation, absorbing harmful elements, and inducing transformation-induced plasticity (TRIP) effects.

**Figure 6:**
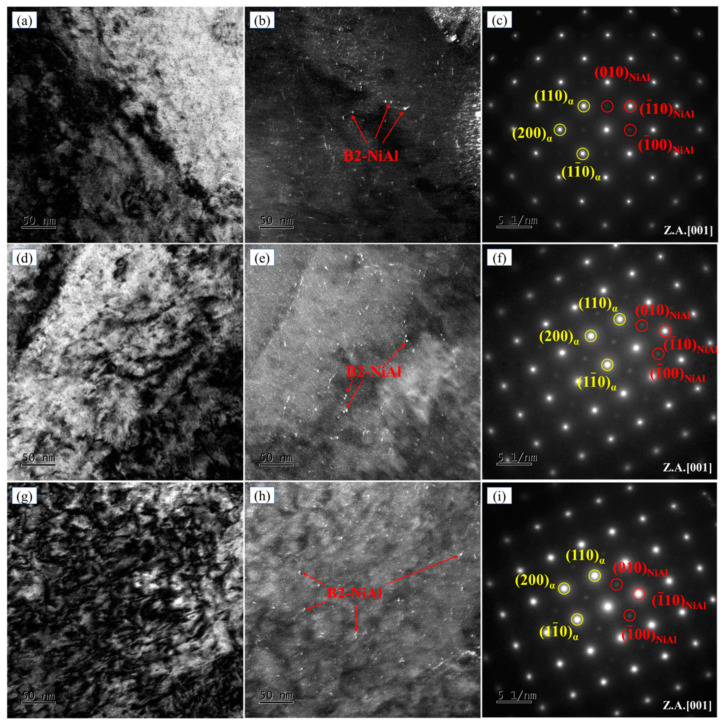
TEM characterization of NiAl phase in experimental steel tempered at different temperatures. Bright-field images: (**a**) 600 °C, (**d**) 620 °C, and (**g**) 640 °C. Dark-field images: (**b**) 600 °C, (**e**) 620 °C, and (**h**) 640 °C. SAED: (**c**) 600 °C, (**f**) 620 °C, and (**i**) 640 °C.

Dark-field images revealed the presence of fine, dispersed spherical precipitates in the tempered specimens (Figure 6b,e,h). SAED patterns from precipitate-containing regions (insets in Figure 6c,f,i) exhibited superlattice lattices. The measured interplanar spacings of the (110) and (200) planes in Figure 6c were 0.2054 nm and 0.1465 nm, respectively. These values closely match the corresponding ferrite reference spacings of 0.2027 nm and 0.1433 nm from the PDF card (ICDD database). Therefore, the bright diffraction spots can be unambiguously indexed to the ferrite phase. The indexing of the dual diffraction patterns confirmed that the bright spots corresponded to the body-centered cubic (BCC) α-Fe matrix, while the faint spots originated from the B2-structured NiAl phase. The crystallographic orientation relationship between α-Fe and NiAl was determined as [001]_α-Fe_//[001]_NiAl_, demonstrating the coherent precipitation of NiAl within the matrix. Tempering between 600 °C and 640 °C promoted NiAl precipitation, with nanoscale spherical particles (<1 nm) being uniformly dispersed in the matrix (Figure 6b,e,h).

TEM characterization of the TMCP-processed and tempered steels (600 °C, 620 °C, 640 °C) demonstrated that nanoscale secondary-phase particles effectively strengthened the matrix during tempering. Concurrently, reversed austenite formed at grain boundaries. Although this soft phase reduced strength, it enhanced toughness through mechanisms such as crack deflection and stress relaxation. Correlation with mechanical property data (Figure 3) suggested that the strengthening effect from precipitates outweighed the softening effect induced by reversed austenite during tempering.

**Figure 7:**
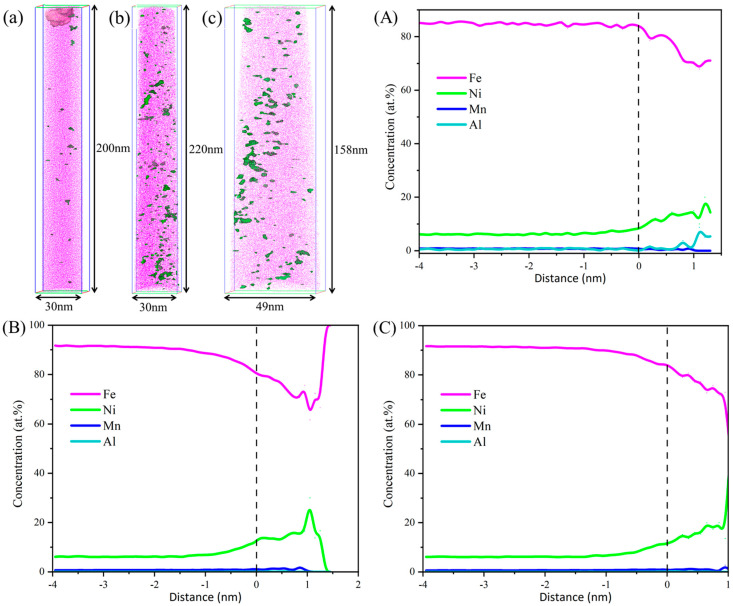
APT characterization of 8.5% (Mn + Ni + Al) iso-concentration surfaces in steel tempered at different temperatures. (**a**) TMCP state; (**A**) Proxigram of TMCP state; (**b**) 600 °C; (**B**) Proxigram of 600 °C; (**c**) 620 °C; (**C**) Proxigram of 620 °C.

To further elucidate the precipitation behavior of nanoscale phases, APT was performed on the TMCP-processed steel and specimens tempered at 600 °C and 620 °C. The NiAl precipitates were identified using 8.5% iso-concentration surfaces for Ni, Mn, and Al, as shown in Figure 7, with quantitative data on the equivalent radius and number density summarized in Table 2. In the TMCP condition (Figure 7a), sparse Ni and Al atom clusters were observed, exhibiting an equivalent radius of 0.67 ± 0.05 nm and a number density of 1.82 × 10^23^/m^3^. The low density and nanometer size of these clusters rendered them undetectable in TEM imaging. After tempering at 600 °C (Figure 7b), the NiAl precipitates grew slightly to 0.78 ± 0.20 nm, while their number density significantly increased to 5.28 × 10^24^/m^3^, confirming enhanced precipitation kinetics at this temperature. For the specimen tempered at 620 °C for 3 h (Figure 7c), the NiAl precipitates maintained a stable size (0.76 ± 0.18 nm) but exhibited a reduced number density of 5.67 × 10^23^/m^3^, which remained higher than that of the TMCP condition (1.82 × 10^23^/m^3^). Compared to the 600 °C tempered steel, the NiAl precipitates in the 620 °C tempered specimen primarily underwent dissolution, leading to a reduced number density, while their size remained nearly constant during the tempering process.

**Figure 8:**
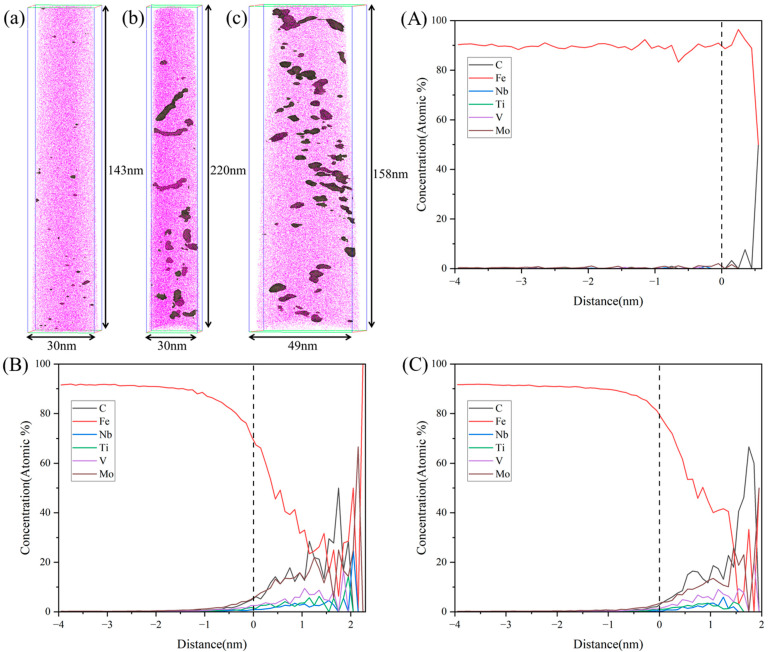
APT characterization of 1.5%C iso-concentration surfaces in steel tempered at different temperatures. (**a**) TMCP state; (**A**) Proxigram of TMCP state; (**b**) 600 °C; (**B**) Proxigram of 600 °C; (**c**) 620 °C; (**C**) Proxigram of 620 °C.

To further confirm the precipitation states of nanoscale carbides, APT was performed on the TMCP-processed steel and specimens tempered at 600 °C and 620 °C. Carbides were highlighted using 1.5% iso-concentration surfaces for carbon atoms, as shown in Figure 8. Under the TMCP conditions, negligible nanoscale carbide precipitation was observed (Figure 8a). However, after tempering at 600 °C and 620 °C, the pronounced segregation of C, Ti, V, Nb, and Mo atoms resulted in clustered regions, indicating the precipitation of (Ti, V, Nb)C and Mo2C carbides within the matrix during tempering. For the 600 °C tempered steel (Figure 8b), the carbides exhibited an equivalent radius of 1.15 ± 0.51 nm and a number density of 1.23 × 1024 m⁻3. In contrast, tempering at 620 °C (Figure 8c) resulted in carbides with an approximate size of 1.01 ± 0.58 nm but a significantly reduced number density of 2.72 × 1023 m⁻3. This indicates that during the tempering process from 600 °C to 620 °C, the size of the nanoscale carbides remained nearly unchanged. However, as the tempering temperature approached the two-phase region, partial dissolution of the carbides occurred, leading to a significant reduction in their number density.

The dispersion of nanoscale precipitates strengthens the matrix primarily by impeding dislocation motion. In the TMCP-processed steel, the limited number of Ni/Al clusters and the absence of carbides contributed minimally to strength. Tempering at 600 °C significantly enhanced strength due to the precipitation of densely distributed nanoscale NiAl phases and carbides (Figure 7b and Figure 8b). However, as the tempering temperature increased to 620 °C, the dissolution of precipitates became dominant. The reduced number density of precipitates (Figure 7c and Figure 8c) led to diminished precipitation strengthening, consequently lowering the yield strength compared to the 600 °C tempered condition.

## 4. Discussion

### 4.1. Strengthening Model of ULCB Steel

The strength evolution of the experimental steel during tempering was governed by two competing factors: (1) softening induced by reduced dislocation density due to the recovery of the bainitic matrix and (2) precipitation strengthening from nanoscale secondary-phase particles. As both dislocation density and the size/number density of precipitates evolved during tempering, the interplay between these mechanisms dictated the yield strength behavior.

To quantitatively assess the impact of tempering on yield strength, theoretical strengthening models [19] were applied to the TMCP-processed and 600 °C tempered specimens. The total yield strength (σ_y_) can be expressed as follows:

(1) $$\sigma y=\sigma_{0}{+\sigma }_{S}+\sigma_{HP}+{{(\sigma }_{d}^{2}+\sigma_{p}^{2})}^{1/2}$$

In this equation, σ_0_ represents the lattice friction stress of pure iron, taken as 87 MPa [20]; σ_S_ denotes the contribution of solid solution strengthening; σ_HP_ corresponds to grain boundary (Hall–Petch) strengthening; σ_d_ accounts for dislocation strengthening; and σ_p_ quantifies precipitation strengthening.

The contribution of the solid-solution strengthening can be expressed as follows [21]:

(2) $$\sigma_{s}={\left(\sum_{i}\beta_{i}^{2}X_{i}\right)}^{1/2}$$

In this equation, β_i_ represents the strengthening coefficient of element i, where β_C_ = 959, β_Mn_ = 540, β_Cr_ = 622, β_Mo_ = 2362, β_V_ = 135, β_Ni_ = 708, and β_Al_ = 196. Xi denotes the atomic percentage of element i.

The contribution of the grain boundary strengthening can be expressed as follows [22]:

(3) $$\sigma_{HP}=Kd^{-1/2}$$

In this equation, K is the Hall–Petch constant, and its value is 0.21 MPa·m^1/2^ [22]. The experimental steel exhibited a bainitic microstructure, in which the effective grain size corresponds to the bainitic lath width. EBSD analysis confirmed that the effective grain sizes were 2.54 μm for the TMCP condition and 2.89 μm for the 600 °C tempered condition.

The increase in shear stress caused by dislocation strengthening can be given as follows [23,24]:

(4) $$\sigma_{d}=\alpha \mathrm{MGb}\rho_{d}^{1/2}$$

**Figure 9:**
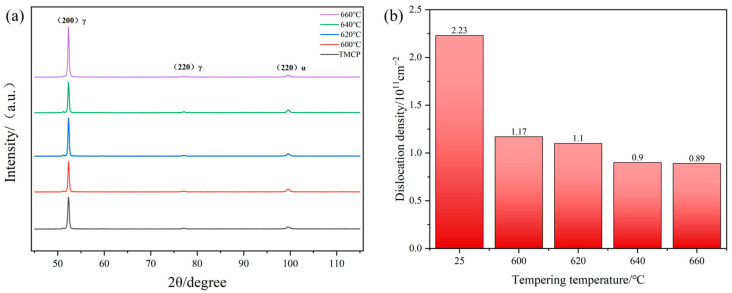
The experimental steel tempered at different temperatures: (**a**) XRD patterns; (**b**) dislocation density.

In this equation, α is the proportionality constant, taken as 0.13; M represents the Taylor factor, set to 2.75 [25]; G denotes the shear modulus, 76 GPa [26]; b is the magnitude of the Burgers vector, 0.248 nm; and ρ_d_ corresponds to the dislocation density. The dislocation density in the experimental steel was determined using XRD, with the results summarized in Figure 9. The data reveal that the dislocation density exhibited a significant reduction after tempering and gradually decreased with increasing tempering temperature.

Precipitation strengthening in the experimental steel is categorized into contributions from carbide precipitates and B2-NiAl phase precipitates. The carbides, acting as hard phases with equivalent radii exceeding 1 nm, are considered to strengthen the matrix via the Orowan bypass mechanism. The Orowan strengthening model is expressed as follows [27]:

(5) $$\sigma_{MC}=0.1132\frac{Gbf^{1/2}}{R}ln(4.032R)$$

In this equation, R represents the average radius of the carbides; f denotes the volume fraction of the carbides, which is obtained for the experimental steel using the following equation [28]:

(6) $$f=4/3\pi R^{3}N$$

In this equation, N represents the number density of nanoparticles.

The NiAl precipitates, with sizes approximating 1 nm, contribute to strengthening via the shearing mechanism [27,29]. Shearing-induced strengthening encompasses four components: order strengthening, modulus mismatch strengthening, coherency strengthening, and chemical strengthening.

(7) $$\sigma_{NiAl}={\left(\sigma_{order}^{2}+\sigma_{modulus}^{2}+\sigma_{coherency}^{2}+\sigma_{chemical}^{2}\right)}^{1/2}$$

The increase in order strengthening caused by the B2-NiAl precipitates can be given by the following:

(8) σorder =Mr32b4rsfπT1/2

In this equation, r is the average antiphase boundary (APB) energy of the NiAl phase (0.5 J/m^2^ [28]); r_s_ represents the average particle radius on the slip plane, calculated as r_s_ = (2/3)^1/2^·R; and T denotes the line tension associated with dislocations, given by T = Gb^2^/2.

The increase in modulus mismatch strengthening caused by the B2-NiAl precipitates can be given by the following:

(9) $$\sigma_{\mathrm{modulus}}=\frac{\mathrm{MGb}}{L}{[1-{\left(\frac{E_{P}}{E_{m}}\right)}^{2}]}^{3/4}$$

In this equation, L is the average inter-precipitate spacing on the slip plane, calculated as L = 0.866/(RN)^1/2^; and Ep and Em represent the dislocation line energies of the precipitate and matrix, respectively, with Ep/Em = 0.987 [28].

The increase in coherency strengthening caused by the B2-NiAl precipitates can be given by the following:

(10) $$\sigma_{\mathrm{coherency}}=4.1G\varepsilon^{3/2}f^{\frac{1}{2}}{\left(\frac{R}{b}\right)}^{1/2}$$

In this equation, ε denotes the constrained lattice misfit parameter, taken as 0.001333 [26]. 

The increase in chemical strengthening caused by the B2-NiAl precipitates can be given by the following:

(11) $$\sigma_{\mathrm{chemical}}=\frac{2M}{bLT^{\frac{1}{2}}}{\left(\gamma_{\mathrm{interfacial}}b\right)}^{3/2}$$

In this equation, $\gamma_{\mathrm{interfacial}}$ is the interfacial energy, set to 0.35 J/m^2^ [26].

Table 3 summarizes the calculated contributions of individual strengthening mechanisms to the yield strength, along with the experimentally measured values. The theoretical calculations align well with the experimental measurements for both the TMCP-processed steel and the 600 °C tempered steel. This consistency demonstrates that tempering effectively enhances the yield strength of the experimental steel by promoting the precipitation of nanoscale precipitates. Specifically, tempering at 600 °C after TMCP induces the formation of densely distributed nanoscale carbides and NiAl precipitates, which significantly improve strength but concurrently reduce the toughness of the steel.

### 4.2. Toughening Characteristics

**Figure 10:**
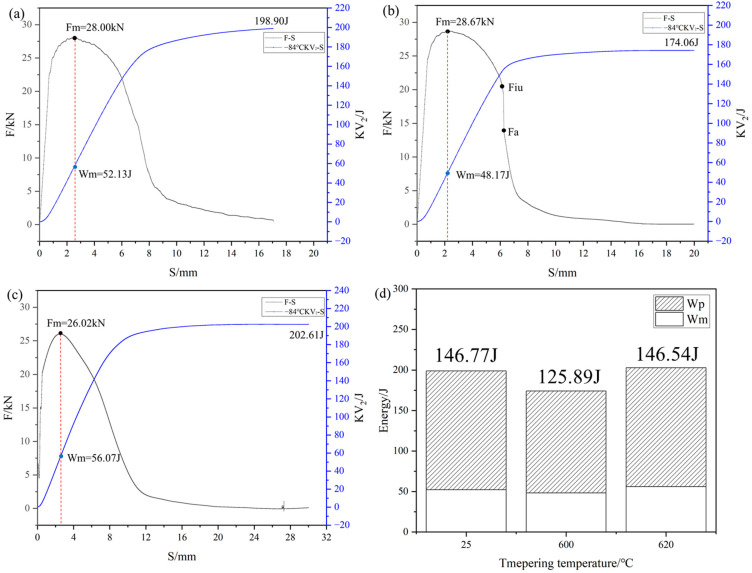
Instrumented impact curves of experimental steel tempered at different temperatures tested at −84 °C: (**a**) TMCP, (**b**) 600 °C, (**c**) 620 °C, and (**d**) crack initiation energy and crack propagation energy.

The instrumented impact test results under different conditions are presented in Figure 10. At −84 °C, the crack initiation energies for the TMCP-processed steel, 600 °C tempered steel, and 620 °C tempered steel were 52.13 J, 48.17 J, and 56.07 J, respectively, showing a slight decrease followed by an increase. In contrast, the crack propagation energies exhibited more pronounced variations of 146.77 J, 125.89 J, and 146.54 J, respectively, following the same trend as the initiation energy. Furthermore, the crack propagation curves transitioned from transverse F-type to longitudinal E-type and back to transverse F-type. These results indicate that the toughness of the steel first decreased and then recovered during tempering.

Under TMCP conditions, the steel exhibited relatively high toughness due to its low strength and absence of precipitates. After tempering at 600 °C, the formation of fine, dispersed NiAl precipitates and nanoscale carbides induced dislocation pile-ups near the precipitates, enhancing strength but deteriorating the toughness of steel. As the tempering temperature increased to 620 °C, the reduced number density of precipitates and increased volume fraction of reversed austenite synergistically contributed to partial toughness recovery.

## 5. Conclusions

Through a combined TMCP and tempering (600–660 °C) approach, a multi-component strengthened ULCB steel incorporating nanoscale second-phase particles were successfully developed. The nanostructural characteristics of the NiAl precipitates were systematically characterized via TEM and APT, elucidating their mechanistic impacts on mechanical properties. The quantitative contributions of solid-solution strengthening, dislocation strengthening, grain boundary strengthening, and precipitation strengthening were calculated based on the mechanical properties and precipitation behaviors of the TMCP-processed and 600 °C tempered steels. The key findings are summarized as follows:

(1) A Mn-Ni-Al-modified ULCB steel was fabricated via TMCP followed by tempering, achieving a yield strength of 797.0–879.5 MPa, a yield ratio of 0.76–0.84, and an impact energy of 172.0–190.3 J at −84 °C.

(2) Tempering at 600–640 °C enhanced the yield strength of the TMCP-processed steel. In the TMCP condition, the strength increment primarily originated from high-density dislocations and grain refinement. Subsequent tempering promoted the precipitation of nanoscale Mo_2_C, (V, Nb, Ti)C carbides and B2-NiAl phases, which dispersed homogeneously in the matrix to further strengthen the steel. However, as the tempering temperature increased, the partial dissolution of these nanoscale precipitates reduced their strengthening contribution.

(3) The precipitation of nanoscale phases during 600–660 °C tempering degraded the impact toughness of the steel. Nevertheless, with tempering temperatures rising from 600 °C to 660 °C, the dissolution of precipitates and the concurrent formation of reversed austenite stabilized the impact toughness at a relatively high level.

## Figures and Tables

**Table 1:** Chemical composition of experimental steel.

Element	C	Al	Si	Mn	Ni	Cr	Mo	V	Nb	Ti	Fe
wt.%	0.044	0.24	0.21	0.68	7.10	0.51	0.61	0.061	0.043	0.020	Bal.
at.%	0.204	0.496	0.417	0.691	6.751	0.547	0.355	0.067	0.026	0.023	Bal

**Table 2:** Equivalent radius and number density of NiAl clusters in steel tempered at different temperatures.

States	R_p_/nm	N_v_/m^−3^
TMCP	0.67 ± 0.05	1.82 × 10^23^
600 °C 3 h	0.78 ± 0.20	5.28 × 10^24^
620 °C 3 h	0.76 ± 0.18	5.67 × 10^23^

**Table 3:** Contributions of strengthening mechanisms to yield strength.

Strength (MPa)	σ_experiment_	σ_model_	σ_0_	σ_S_	σ_HP_	σ_d_	σ_MC_	σ_order_	σ_modulus_	σ_coherency_	σ_chemical_
TMCP	697.0	701.8	87	161	132	318	0	32	43	1	5
600 °C	879.5	856	87	161	124	230	252	237	248	8	27

## Data Availability

The original contributions presented in this study are included in the article. Further inquiries can be directed to the corresponding author.

## References

[1] Zha X., Xiong Y., Zhou T., Ren Y., Hei P., Zhai Z., Kömi J., Huttula M., Cao W. (2020). Impacts of Stress Relief Treatments on Microstructure, Mechanical and Corrosion Properties of Metal Active-Gas Welding Joint of 2205 Duplex Stainless Steel. Materials.

[2] Imanian Ghazanlou S., Mobasher Amini A., Carrier F.-A., Sarkar D.K., Rehman K., Javidani M. (2024). Study of the Microstructure and Mechanical Properties of Steel Grades for Ship Hull Construction. Materials.

[3] Yu L., Guo W., Cao C., Li M., Wu Z., Wang T., Chen H., Pan X. (2024). Experimental Study on the Fatigue Crack Propagation Rate of 925A Steel for a Ship Rudder System. Materials.

[4] Hao X. (2022). Effect of Isothermal Quenching near Ms Point on Microstructure and Properties of Medium-Low Carbon Steel. Master’s Thesis.

[5] Su C. (2022). Study on Microstructure and Plasticity-Toughness of 1000 MPa Grade Hot-Rolled Ultra-High Strength Steel. Master’s Thesis.

[6] Han C., Liu Q., Cai Z., Sun Q., Huo X., Fan M., He Y., Li K., Pan J. (2022). Effect of tempering heat treatment on the microstructure and impact toughness of a Ni-Cr-Mo-V steel weld metal. Mater. Sci. Eng. A Struct. Mater. Prop. Misrostructure Process..

[7] Li Z., Chai F., Luo X., Zhang Z., Yang C., Su H. (2020). Effect of aging temperature on mechanical properties of Cu precipitation strengthened ultra-high strength marine steel. Mater. Rep..

[8] Li Z., Chai F., Yang C., Luo X., Yang L., Su H. (2018). Effect of quenching process on mechanical properties of UHS marine steel. Chin. J. Mater. Res..

[9] Xiao Y., Peng B., Fan A., Jiang S., Wang X. (2023). Complex precipitation behavior and strengthening mechanisms of Fe-Ni-Al ultra-high strength dual-phase steel. J. Iron Steel Res..

[10] Jiao Z., Liu J. (2011). Research and development of novel nano-strengthened ultra-high strength steels. Mater. China.

[11] Gao J., Jiang S., Zhang H., Huang Y., Guan D., Xu Y., Guan S., Bendersky L.A., Davydov A.V., Wu Y. (2021). Facile route to bulk ultrafine-grain steelsfor high strength and ductility. Nature.

[12] Zhang X. (2024). Characterization of NiAl Nanoprecipitates and Strengthening-Toughening Mechanisms in Ultra-High Strength Hull Steel. Ph.D. Thesis.

[13] Li Z., Chai F., Yang L., Luo X., Yang C. (2020). Mechanical properties and nanoparticles precipitation behavior of multi-component ultra high strength steel. Mater. Des..

[14] Shikanai N., Kagawa H., Kurihara M. (1992). Influence of Microstructure on Yielding Behavior of Heavy Gauge High Strength Steel Plates. ISIJ Int..

[15] Tong M.W., Venkatsurya P.K.C., Zhou W.H., Misra R.D.K., Guo B., Zhang K.G., Fan W. (2014). Structure-mechanical property relationship in a high strength microalloyed steel with low yield ratio: The effect of tempering temperature. Mater. Sci. Eng. A.

[16] (2021). Metallic Materials—Tensile Testing—Part 1: Method of Test at Room Temperature.

[17] (2020). Metallic Materials—Charpy Pendulum Impact Test Method.

[18] Miller M.K. (2012). Atom Probe Tomography: Analysis at the Atomic Level.

[19] Chen J., Lv M.-Y., Tang S., Liu Z.-Y., Wang G.-D. (2018). Influence of cooling paths on microstructural characteristics and precipitation behaviors in a low carbon V-Ti microalloyed steel. Mater. Sci. Eng. A.

[20] Yong Q.L. (2006). Secondary Phases in Steels.

[21] Galindo-Nava E.I., Rainforth W.M., Rivera-Diaz-Del-Castillo P.E.J. (2016). Predicting microstructure and strength of maraging steels:Elemental optimisation. Acta Mater..

[22] Shibata A., Nagoshi T., Sone M., Morito S., Higo Y. (2010). Evaluation of the block boundary and sub-block boundary strengths of ferrous lath martensite using a microbending test. Mater. Sci. Eng. A.

[23] Huang M., Rivera-Díaz-del-Castillo P.E.J., Bouaziz O., van der Zwaag S. (2009). Modelling strength and ductility of ultrafine grained BCC and FCC alloys using irreversible thermodynamics. Mater. Sci. Technol..

[24] Kamikawa N., Sato K., Miyamoto G., Murayama M., Sekido N., Tsuzaki K., Furuhara T. (2015). Stress-strain behavior of ferrite and bainite with nano-precipitation in low carbon steels. Acta Mater..

[25] Keh A.S. (1965). Work hardening and deformation sub-structure in iron single crystals deformed in tension at 298k. Philos. Mag..

[26] Zhou T., Faleskog J., Babu R.P., Odqvist J., Yu H., Hedström P. (2019). Exploring the relationship between the microstructure and strength of fresh and tempered martensite in a maraging stainless steel Fe-15Cr-5Ni. Mater. Sci. Eng. A.

[27] Xu S.S., Zhao Y., Chen D., Sun L.W., Chen L., Tong X., Liu C.T., Zhang Z.W. (2019). Nanoscale precipitation and its influence on strengthening mechanisms in an ultra-high strength low-carbon steel. Int. J. Plast..

[28] Jiao Z.B., Luan J.H., Miller M.K., Yu C.Y., Liu C.T. (2015). Effects of Mn partitioning on nanoscale precipitation and mechanical properties of ferritic steels strengthened by NiAl nanoparticles. Acta Mater..

[29] Jiao Z.B., Luan J.H., Zhang Z.W., Miller M.K., Liu C.T. (2014). High-strength steels hardened mainly by nanoscale NiAl precipitates. Scr. Mater..

